# Adiponectin Suppresses Metastasis of Nasopharyngeal Carcinoma through Blocking the Activation of NF-κB and STAT3 Signaling

**DOI:** 10.3390/ijms232112729

**Published:** 2022-10-22

**Authors:** Zongmeng Zhang, Jinlin Du, Qihua Xu, Chaofeng Xing, Yuyu Li, Sujin Zhou, Zhenggang Zhao, Yunping Mu, Zijian (Allan) Zhao, Sumei Cao, Fanghong Li

**Affiliations:** 1 The School of Biomedical and Pharmaceutical Sciences, Guangdong University of Technology, Guangzhou 510006, China; 2 Department of Epidemiology and Health Statistics, School of Public Health, Guangdong Medical University, Dongguan 523808, China; 3 Department of Cancer Prevention Research, Sun Yat-sen University Cancer Center, Guangzhou 510060, China

**Keywords:** adiponectin, nasopharyngeal carcinoma, metastasis, STAT3, NF-κB

## Abstract

Adiponectin is an adipocytokine with anti-inflammatory and anticancer properties. Our previous study has shown that blood adiponectin levels were inversely correlated to the risk of nasopharyngeal carcinoma (NPC), and that adiponectin could directly suppress the proliferation of NPC cells. However, the effect of adiponectin on NPC metastasis remains unknown. Here, we revealed in clinical studies that serum adiponectin level was inversely correlated with tumor stage, recurrence, and metastasis in NPC patients, and that low serum adiponectin level also correlates with poor metastasis-free survival. Coculture with recombinant adiponectin suppressed the migration and invasion of NPC cells as well as epithelial–mesenchymal transition (EMT). In addition, recombinant adiponectin dampened the activation of NF-κB and STAT3 signaling pathways induced by adipocyte-derived proinflammatory factors such as leptin, IL-6, and TNF-α. Pharmacological activation of adiponectin receptor through its specific agonist, AdipoRon, largely stalled the metastasis of NPC cells. Taken together, these findings demonstrated that adiponectin could not only regulate metabolism and inhibit cancer growth, but also suppress the metastasis of NPC. Pharmacological activation of adiponectin receptor may be a promising therapeutic strategy to stall NPC metastasis and extend patients’ survival.

## 1. Introduction

Nasopharyngeal carcinoma (NPC) is a malignant tumor originating from the epithelium of the nasopharyngeal mucosa [1]. Global incidence is mainly concentrated in East Asia and Southeast Asia [2,3]. Through the screening of Epstein–Barr virus (EBV) and early treatment, the patients’ survival has been significantly improved [4,5]. However, local recurrence and distant metastasis are still the leading causes of death among NPC patients [6]. Chronic inflammation participates in the development, recurrence, and metastasis of malignant tumors, and is one of the important characteristics of NPC [7,8,9,10]. In the tumor microenvironment, chronic inflammation can induce the enrichment of inflammatory factors and promotes tumor recurrence, chemotherapy resistance, angiogenesis, invasion, and metastasis [11,12].

Adipose tissue is not only involved in energy storage, but also, as a major endocrine organ, participates in the formation of the tumor microenvironment by secreting various hormones and cytokines, such as leptin, adiponectin, and tumor necrosis factor alpha (TNF-α) [13]. Abundant studies have found that adipocytokines play important roles in tumor invasion and metastasis, among which resistin and leptin have been shown to promote metastasis in a variety of tumor models [14,15,16]. Several other cytokines, such as IL-2, TNF-α, and IL-6, were also reported to be associated with pathological grades, treatment response, and prognosis in NPC [17,18,19].

Traditionally, adiponectin is known to have an important regulatory effect on glucose and lipid metabolism, alleviate insulin resistance, and lessen the development of inflammation and atherosclerosis [20,21,22,23]. In the tumor microenvironment, adiponectin inhibits tumorigenesis and progression by antagonizing proinflammatory signaling [24,25,26,27]. In patients with malignancies, the level of circulating adiponectin is negatively correlated with tumor metastasis [28,29,30]. Our previous studies have also found that adiponectin inhibits the proliferation of endometrial cancer cells [31], and null mutation of adiponectin increases the occurrence of endometrial cancer in the PTEN heterozygotic mutant mouse model [32]. Furthermore, our recent study has shown that serum adiponectin level was negatively associated with the risk of NPC and could suppress the growth of NPC through activating the AMPK signaling pathway [33]. In this study, we aim to investigate the association between adiponectin level with clinicopathological status in NPC patients, the effects of adiponectin on migration and invasion in NPC cells, and to examine the underlying mechanisms.

## 2. Results

### 2.1. Association of Serum Adiponectin Level with Clinicopathological Characteristics

Our previous study has demonstrated that serum adiponectin level was inversely associated with the risk of NPC [33]. In analyzing a clinical cohort of 106 NPC patients (Table 1) with complete records of clinicopathological characteristics and blood adiponectin concentrations, we found that median blood adiponectin level was lower in patients with high-grade (stage III and IV) tumors compared with that in patients with low-grade tumors (stage I and II) (2.06 vs. 3.87 μg/mL, *p* = 0.019), and that similar findings were also observed in recurrent patients vs. nonrecurrent patients (1.05 vs. 2.28 μg/mL, *p* = 0.031) (Table 1). Importantly, the patients with metastasis displayed significantly lower blood adiponectin levels comparted with the patients with no metastasis (1.57 vs. 3.05 μg/mL, *p* = 0.001) (Table 1).

**Figure 1:**
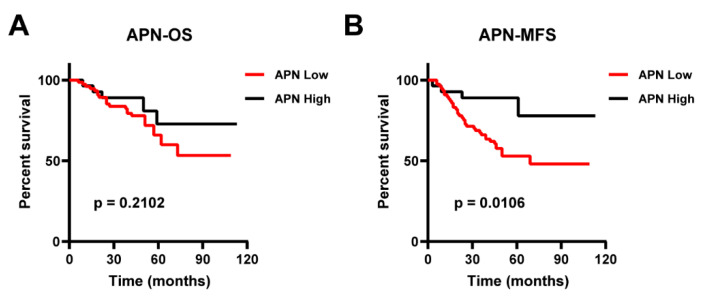
The association of serum adiponectin levels with survival in NPC patients. Kaplan–Meier curves were used to illustrate several patients. (**A**) Overall survival and (**B**) metastasis-free survival were analyzed according to serum adiponectin levels based on a cutoff of 8.00 μg/mL in NPC patients (*n* = 106). *p*-values were determined by the log-rank test.

We further evaluated the association of blood adiponectin levels with the survival rates of NPC patients by Kaplan–Meier estimates. All subjects were divided into two groups based on serum adiponectin levels, with the median value as the cutoff (8.00 μg/mL). Although circulating adiponectin level was not significantly associated with the overall survival (OS) (*p* = 0.2102) (Figure 1A), the high adiponectin concentration group clearly showed better prognosis in metastasis-free survival (MFS) compared with the low adiponectin concentration group (*p* = 0.0106, Figure 1B), and such result was also confirmed in a univariable analysis including tumor grade, recurrence, and blood adiponectin level (Hazard-Ratio (HR): 0.282; 95% CI: 0.100–0.796; *p* = 0.005) (Table 2). Furthermore, in a multivariable adjusted model including age, tumor grade, family history, VCA-IgA, EBNA1/IgA, and BMI in NPC patients, circulating adiponectin remained an independent predictor of MFS (HR, 0.245; 95% CI: 0.0.080–0.745; *p* = 0.013) (Table 2).

### 2.2. Adiponectin Inhibits Migration and Invasion of NPC Cell

**Figure 2:**
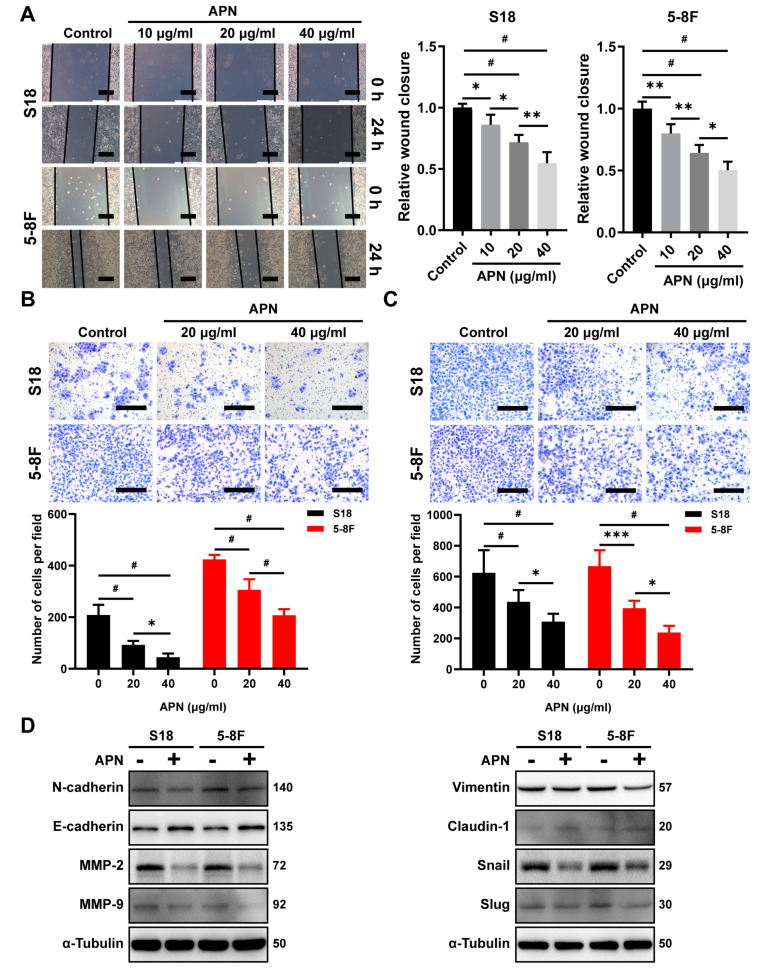
Adiponectin suppresses NPC cells invasion and migration. (**A**) Wound healing assay of S18 and 5-8F cells pretreated with adiponectin (0, 10, 20, or 40 μg/mL) for 24 h. Scale bar: 200 μm. Graphs show the relative wound closure. (**B**) Invasion of S18 and 5-8F cells were evaluated by Transwell invasion assay covered with Matrilgel after treatment with various dosing of adiponectin for 24 h. Scale bar: 200 μm. Graphs show the relative number of invasion cells. (**C**) Migration of S18 and 5-8F cells were evaluated by Transwell migration assay after treatment with various doses of adiponectin for 24 h. Scale bar: 200 μm. Graphs show the relative number of migration cells. (**D**) Western blot analysis of E-cadherin, N-cadherin, Slug, Snail, Vimentin, Claudin-1, MMP-2, and MMP-9 in S18 and 5-8F cells after treatment with 40 μg/mL adiponectin for 48 h. Results are presented as mean ± SD of three independent experiments performed in triplicate. * *p* < 0.05, ** *p* < 0.01, *** *p* < 0.001, ^#^ *p* < 0.0001.

As serum adiponectin level was significantly inversely correlated with metastasis in NPC patients (Table 1), wound healing and Transwell assays were performed to examine whether adiponectin would impact the migration and invasion of NPC cells. Wound healing assay revealed that adiponectin could significantly suppress the migration of S18 and 5-8F cells in a dose-dependent manner (Figure 2A). Furthermore, adiponectin treatment suppressed the invasion of S18 and 5-8F cells in a dose-dependent manner through Transwell assays with Matrigel (Figure 2B). Consistently, Transwell migration assay showed that adiponectin decreased the migration of S18 and 5-8F cells (Figure 2C). Epithelial–mesenchymal transition (EMT) was involved in the invasion and migration of tumor cells; in analyzing the expression of EMT proteins that are critical for initiating metastasis, we found that adiponectin treatment elevated the expression of E-cadherin and Claudin-1 while decreasing the level of N-cadherin, vimentin, MMP-2, MMP-9, Snail, and Slug in S18 and 5-8F cells (Figure 2D), further supporting that adiponectin can impair NPC cell migration and invasion by inhibiting EMT.

### 2.3. Adiponectin Blocks the Migration and Invasion Induced by IL-6, TNF-α, and Leptin Treatment

**Figure 3:**
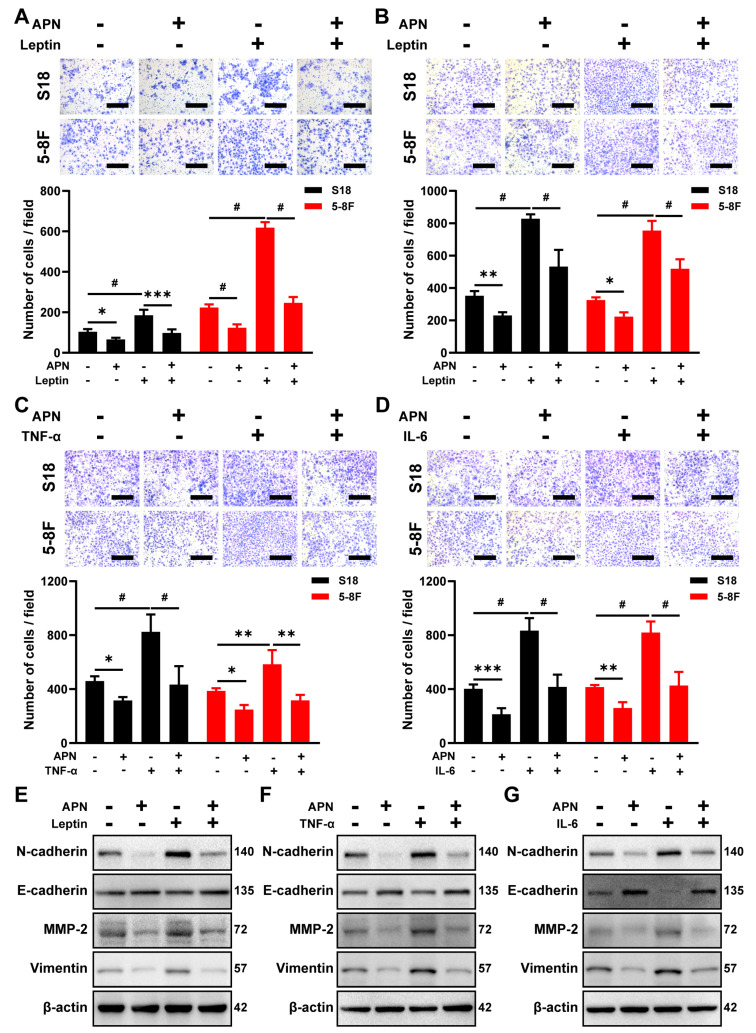
Adiponectin blocks leptin, IL-6, or TNF-α-induced NPC cell invasion and migration. Transwell migration (**A**) and invasion (**B**) assays in S18 and 5-8F cells pretreated with 40 μg/mL adiponectin for 2 h before treatment with 50 ng/mL leptin. Scale bar: 200 μm. Graphs show the relative number of cells. Transwell migration assay in S18 and 5-8F cells pretreated with 40 μg/mL adiponectin for 2 h before treatment with 50 ng/mL TNF-α (**C**) or 50 ng/mL IL-6 (**D**). Scale bar: 200 μm. Graphs show the relative number of migration cells. (**E**–**G**) Western blot analysis of E-cadherin, N-cadherin, MMP-2, and Vimentin in 5-8F cells pretreated with 40 μg/mL adiponectin in the presence or absence of leptin (50 ng/mL), TNF-α (50 ng/mL), or IL-6 (50 ng/mL) for 48 h. Results are presented as mean ± SD of three independent experiments performed in triplicate. * *p* < 0.05, ** *p* < 0.01, *** *p* < 0.001, ^#^ *p* < 0.0001.

Previous studies have demonstrated that some of the proinflammatory cytokines, such as leptin, IL-6, and TNF-α, in the tumor microenvironment could promote tumor progression [12,13]. Consistent with those reports, leptin promoted NPC cell invasion and migration in NPC cells (Figure 3A,B), and IL-6 and TNF-α also significantly induced migration in NPC cells (Figure 3C,D). To explore whether adiponectin could prevent these prometastatic effects, NPC cells were pretreated with adiponectin. Coincubation with adiponectin blocked leptin-induced NPC cell invasion and migration (Figure 3A,B), as well as IL-6- or TNF-α-stimulated cellular migration (Figure 3C,D). Although leptin, IL-6, or TNF-α could promote EMT, as evident in the decreased expression of E-cadherin and elevated expression of N-cadherin, MMP-2, and Vimentin in 5-8F cells (Figure 3E–G), in contrast, cotreatment with adiponectin significantly neutralized these effects, further indicating that adiponectin can block proinflammatory cytokine-induced migration and invasion, possibly through reversing EMT processes in NPC cells.

### 2.4. Adiponectin Inhibits the Activation of NF-κB and STAT3 Signaling Pathways

**Figure 4:**
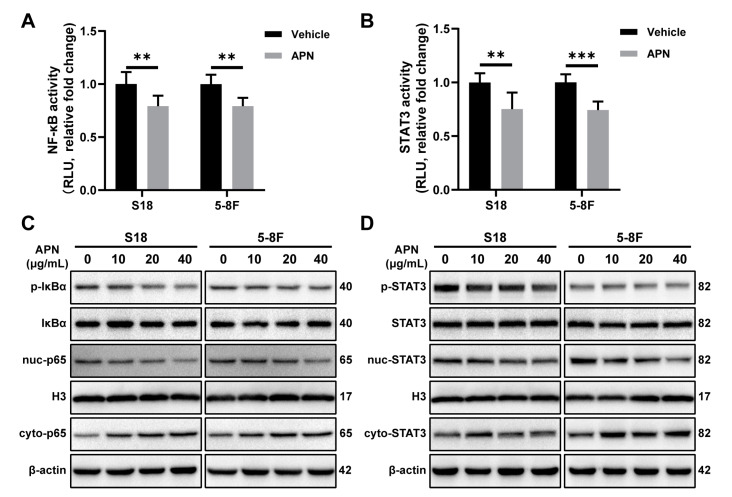
Adiponectin inhibits NF-κB and STAT3 activation in NPC cells. Luciferase reporter assay of NF-κB (**A**) and STAT3 (**B**) in S18 and 5-8F cells after treatment with 40 μg/mL adiponectin for 3 h. (**C**) Western blot analysis of p-IκBα, cyto-p65, and nuc-p65 in S18 and 5-8F cells after treatment with various doses of adiponectinfor 6 h. (**D**) Western blot analysis of p-STAT3, cyto-STAT3, and nuc-STAT3 in S18 and 5-8F cells after treatment with various doses of adiponectin for 6 h. Results are presented as mean ± SD of three independent experiments performed in triplicate. ** *p* < 0.01, *** *p* < 0.001.

Previous studies have revealed that NF-κB and STAT3 signaling pathways are intimately involved in EMT [34,35]. To examine the effect of adiponectin on the transcriptional activity of NF-κB and STAT3, the plasmid carrying NF-κB or STAT3 response element luciferase reporter gene was transfected into NPC cells. Through detecting a luciferase signal, we found that adiponectin treatment decreased the transcriptional activity of NF-κB and STAT3 (Figure 4A,B). Adiponectin treatment significantly decreased the phosphorylation of IκBα (p-IκBα), a major mediator of NF-κB signaling. Moreover, the nuclear translocation of p65 was blocked after coincubation of adiponectin with NPC cells (Figure 4C). Under the same condition, we also found that adiponectin inhibited STAT3 phosphorylation and nuclear translocation (Figure 4D). 

Numerous studies have indicated leptin, IL-6, and TNF-α could enhance the metastatic properties of tumor cells by activating of NF-KB or STAT3 signal pathway [16,34,35]. To confirm whether adiponectin blocking NF-KB or STAT3 signal activation derived from leptin, IL-6, and TNF-α in NPC cell, we examined the NF-κB and STAT3 activity in S18 and 5-8F cells cotreated with adiponectin. The results showed that adiponectin-blocked leptin induced the activation of NF-κB and STAT3 signaling activity, as measured by the luciferase reporter assays (Figure S1A,B). Furthermore, adiponectin-blocked leptin induced the phosphorylation of STAT3 and p65 (Figure S1C,D), and immunofluorescence staining also showed that adiponectin inhibited the nuclear translocation of leptin-induced p65 and STAT3 in 5-8F cells (Figure S1E,F). Under the same condition, luciferase reporter assays showed adiponectin suppressed TNF-α-induced NF-κB and IL-6-induced STAT3 signaling activity (Figure S2A,B). In addition, adiponectin-blocked TNF-α-induced p65 phosphorylation and IL-6-induced STAT3 phosphorylation (Figure S2C,D). Correspondingly, immunofluorescence staining showed that adiponectin inhibited the nuclear translocation of TNF-α-induced p65 and IL-6-induced STAT3 in 5-8F cells (Figure S2E,F). Taken together, these results suggested that adiponectin suppressed NPC cell migration and invasion, possibly by blocking NF-κB and STAT3 signaling pathways. 

### 2.5. AdipoR1 Mediates the Inhibitory Effect of Adiponectin on Migration

**Figure 5:**
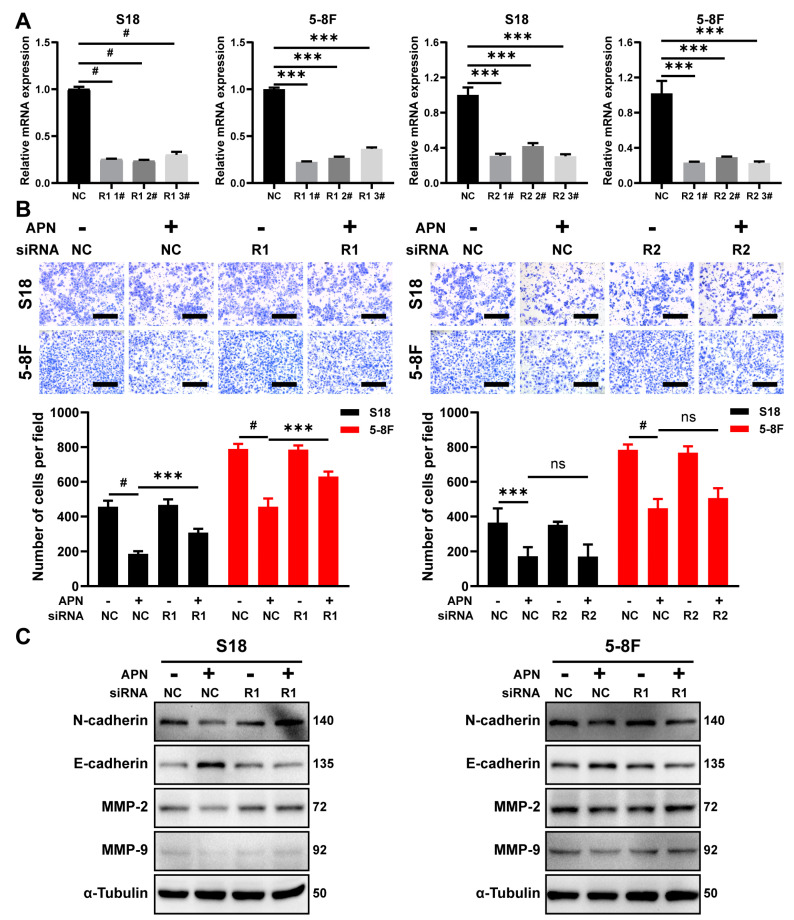
AdipoR1 mediates the metastasis inhibitory effect of adiponectin in NPC cells. (**A**) The mRNA expression of AdipoR1 and AdipoR2 was measured with qRT-PCR in S18 and 5-8F cells after transfected with AdipoR1 or AdipoR2 siRNA. (**B**) Transwell migration assay in S18 and 5-8F cells treated with 40 μg/mL adiponectin for 24 h after transfected with AdipoR1 or AdipoR2 siRNA. Scale bar: 200 μm. Graphs show the relative number of migration cells. (**C**) Western blot analysis of E-cadherin, N-cadherin, MMP-9, and MMP-2 in S18 and 5-8F cells treated with 40 μg/mL adiponectin for 48 h after transfected with AdipoR1 siRNA. Results are presented as mean ± SD of three independent experiments performed in triplicate. *** *p* < 0.001, ^#^ *p* < 0.0001.

To explore the role of AdipoR1 and AdipoR2 in adiponectin-mediated regulation of EMT, siRNAs were transfected to knockdown AdipoR1 or AdipoR2 expression in S18 and 5-8F cells (Figure 5A). We found that knockdown AdipoR1 expression reduced the inhibitory effect of adiponectin on migration (Figure 5B). Knockdown AdipoR2 expression did not have the same effect. Under the same condition, knockdown of AdipoR1 expression prevented the adiponectin-induced upregulation expression of E-cadherin and downregulation expression of N-cadherin, MMP-2, and MMP-9 in NPC cells (Figure 5C). These results demonstrated that AdipoR1 mediated the adiponectin-associated signaling pathways to regulate EMT in NPC cells.

### 2.6. AdipoRon Inhibits NPC Cell Invasion and Migration In Vitro

**Figure 6:**
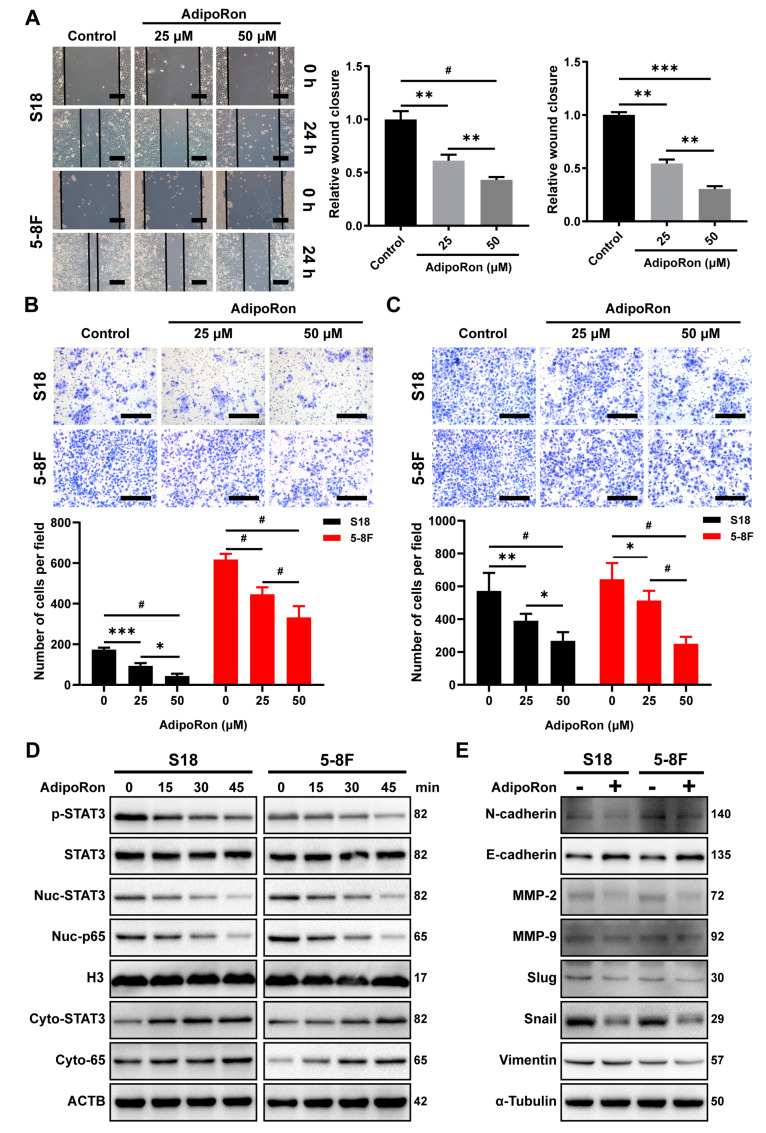
AdipoRon inhibits NPC cell migration and invasion in vitro. (**A**) Wound healing assay of S18 and 5-8F cells treated with AdipoRon (0, 25, or 50 μM) for 24 h. Scale bar: 200 μm. Graphs show the relative wound closure. Transwell (**B**) invasion and (**C**) migration of S18, 5-8F cells after treatment with AdipoRon (0, 25, or 50 μM) for 24 h. Scale bar: 200 μm. Graphs show the relative number of cells. (**D**) Western blot analysis of p-STAT3, cyto-STAT3, nuc-STAT3, cyto-p65, and nuc-p65 in S18 and 5-8F cells after treatment with 50 μM AdipoRon for the indicated time period. (**E**) Western blot analysis of E-cadherin, N-cadherin, Slug, Snail, Vimentin, MMP-2, and MMP-9 in S18 and 5-8F cells after the treatment with 50 μM AdipoRon for 24 h. Results are presented as mean ± SD of three independent experiments performed in triplicate. * *p* < 0.05, ** *p* < 0.01, *** *p* < 0.001, ^#^ *p* < 0.0001.

AdipoRon, a small molecule agonist of adiponectin receptors, was shown to ameliorate type 2 diabetes and cognitive dysfunction of Alzheimer’s disease [36,37]. To explore the effect of AdipoRon in NPC cells’ migration and invasion, we administered AdipoRon to NPC cells and assessed them for wound healing and Transwell assays. Treatment with AdipoRon significantly suppressed the wound healing, migration, and invasion activities of S18 and 5-8F cells in a dose-dependent manner (Figure 6A–C). Next, we examined STAT3 and p65 signaling and found that AdipoRon significantly inhibited STAT3 phosphorylation and nuclear translocation, as well as significantly inhibited p65 nuclear aggregation (Figure 6D). Correspondingly, detection of EMT-related proteins found that AdipoRon significantly increased the expression of E-cadherin and reduced the protein levels of N-cadherin, MMP-9, MMP-2, Snail, Slug, and Vimentin (Figure 6E). Collectively, these results demonstrate that the adiponectin receptor agonist, AdipoRon, can inhibit NPC cells, migration and invasion in vitro.

### 2.7. AdipoRon Inhibits Metastasis of NPC In Vivo

**Figure 7:**
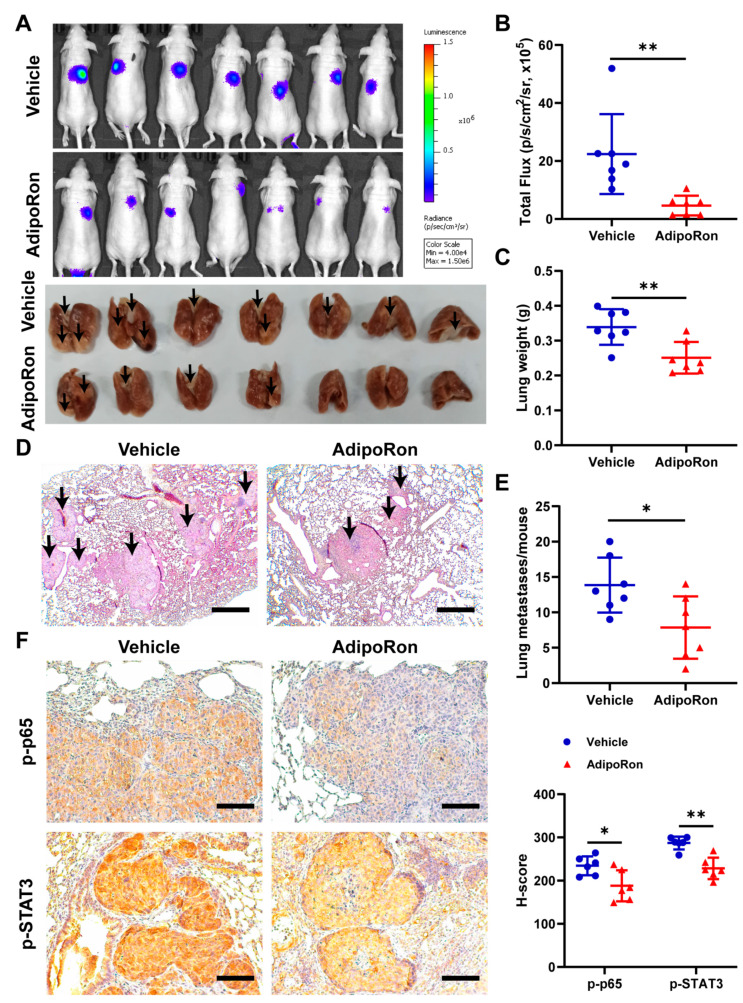
AdipoRon inhibits NPC tumor metastasis in vivo. (**A**) Bioluminescent (upper) and lung (lower) images from 5-8F-luc cells’ lung metastasis model after oral administration of AdipoRon (50 mg/kg/d) or vehicle (*n* = 7 per group). The black arrows indicate metastatic tumors. (**B**) Quantification of in vivo bioluminescent images (photon/s of lung region). (**C**) Wet lungs were counted and summarized. (**D**) Representative H and E staining of lung section. The black arrows indicate metastatic tumors. (**E**) The number of lung metastases were counted and summarized. Scale bar: 200 μm. (**F**) Immunohistochemistry of p-p65 and p-STAT3 protein levels in metastatic 5-8F-luc cells from the lung (*n* = 6). Scale bar: 100 μm. Quantification of protein expression in metastatic 5-8F-luccells. Results are presented as mean ± SD of three independent experiments performed in triplicate. * *p* < 0.05, ** *p* < 0.01.

To understand the effects of adiponectin on metastasis in vivo, we established a lung metastasis model by intravenously injecting 5-8F cells overexpressing a luciferase reporter gene (5-8F-luc) into the nude mice and then treated the mice with either AdipoRon (50 mg/kg/day) or vehicle. There were weaker luciferase signals in lungs of mice treated with AdipoRon compared with the vehicle group (Figure 7A,B). In addition, the AdipoRon-treated group showed less wet weight of the lungs (Figure 7C) and reduced number of metastatic nodules as compared to the vehicle group (Figure 7D,E). Immunohistochemical staining showed that treatment with AdipoRon significantly decreased the levels of p-p65 and p-STAT3 (Figure 7F), further validating the inhibitory effect of adiponectin, likely through the NF-kB and STAT3 pathways, on NPC tumor metastasis.

## 3. Discussion

In this study, we have reported that blood adiponectin level was inversely associated with tumor stage, recurrence, and metastasis in the analysis of a clinical cohort of 106 NPC patients. Importantly, circulating adiponectin level was positively correlated with metastasis-free survival (MFS). This newly identified relationship is completely independent of other factors, such as age, EBV infection status, and family history, suggesting an independent regulation of NPC metastasis.

In corroborating the outcomes of clinical associations, we further established the effects, as well as the underlying mechanisms, of adiponectin on human NPC metastasis. Coincubation with adiponectin or adiponectin receptor agonist suppressed the migration and invasion of human NPC cells by blocking the EMT process. Importantly, we also confirmed that pharmacological activation of adiponectin receptors through a specific agonist, AdipoRon, could inhibit the metastasis of NPC in lung metastasis models. Previous studies have demonstrated that proinflammatory cytokines, such as leptin, TNF-α, and IL-6, promote the migration and invasion of NPC cells [38,39]. Cotreatment with adiponectin almost completely negated the migration and invasion of NPC cells induced by these proinflammatory cytokines. Taken together, these results unequivocally mirrored the clinical observations in the NPC cohort and solidified the concept that adiponectin not only directly inhibits the metastasis of NPC, but also antagonizes the promigration effect induced by proinflammatory factors such as leptin, TNF-α, and IL-6.

Mechanistically, multiple signaling pathways are known to be involved in the regulation EMT and metastasis of malignant cells, including the activation of NF-κB and STAT3 [34,35]. The activation of NF-κB and STAT3 plays important roles in promoting EMT and metastasis and is positively associated with poor prognosis of NPC [40,41,42,43,44]. Our findings here showed that recombinant adiponectin or specific adiponectin receptor agonist, AdipoRon, could block NF-κB and STAT3 activation signaling (including leptin-, IL-6- or TNF-α-induced activation of STAT3 and NF-κB) in NPC cells. A previous work has also reported that pharmacological activation of adiponectin receptor with AdipoRon could attenuate leptin-induced pancreatic tumor growth through blocking pSTAT3 signaling [45]. Thus, AdipoRon can antagonize proinflammatory factor-induced NPC cell growth and metastasis signals in the tumor microenvironment.

Adiponectin can not only directly target the prometastatic signaling of tumor cells, but also inhibit tumor angiogenesis. A previous study has shown that adiponectin inhibits liver tumor angiogenesis through decreasing the activity of the ROCK/IP10/VEGF signaling pathway in an orthotopic liver tumor model [24]. Oral administration of adiponectin receptor agonist AdipoRon has been shown to suppress subcutaneous tumors’ angiogenesis of pancreatic cancer [46]. In our previous study, we also found that AdipoRon significantly inhibited angiogenesis in an NPC cell subcutaneous xenograft model [33]. Furthermore, adiponectin can inhibit angiogenesis by inducing apoptosis of endothelial cells in a murine fibrosarcoma subcutaneous xenograft model [47]. Other studies have found that adiponectin interferes with tumor angiogenesis by regulating tumor-associated macrophage infiltration [48,49]. These studies indicate that adiponectin may also go through the regulation of tumor angiogenesis in NPC models.

## 4. Materials and Methods

### 4.1. Study Designs and Participants

The serum samples of 106 patients with NPC were consecutively collected from the serum bank of Sun Yat-sen University Cancer Center (SYSUCC). The patients were selected based on the criteria as previously described [8]. The TNM staging for patients with NPC was defined according to the staging system described in the seventh edition of Union for International Cancer Control (UICC), and NPCs were classified by the World Health Organization (WHO) classification. All diagnoses of NPC were proven by biopsy. This study was approved by the Institutional Review Board of Sun Yat-sen University Cancer Center (SYSUCC) (NO. YP2009051).

### 4.2. Blood Collection, Detection, and Statistical Analysis

Blood samples were collected in an inert separation gel coagulation tube after overnight fasting. After centrifugation at 2000× *g* for 20 min at 4 °C, the obtained serum was separated and stored at −80 °C until analysis. Circulating adiponectin level was measured using a Milliplex map kit by the Luminex 200TM instrument (Millipore, Billerica, MA, USA) in the Laboratory of Cancer Prevention and Control Center of Sun Yat-sen University (Guangzhou, China). The measurement procedure is strictly in accordance with the standard steps of the manufacturer’s kit. 

The differences of circulating adiponectin concentration among NPC patients with different clinical characteristics were analyzed by Mann–Whitney U test, analysis of variance for continuous variables, and the χ2 test for categorical variables. The metastasis-free survival (MFS) was calculated from the date of treatment to the date of metastasis or death from NPC. MFS and overall survival (OS) were determined using the Kaplan–Meier method and log-rank test. In the analysis of risk factors for the OS and MFS in NPC patients, we tested the following variables obtained at the time of entry in univariate and multivariate Cox proportional hazard regression analysis: gender, age, grade, VCA-IgA, EBNA1/IgA, family history, recurrence, body mass index (BMI), and serum adiponectin level. Statistical analysis was performed using SAS statistical software version 9.4 (SAS Institute, Cary, NC, USA). All hypothesis tests were two-sided test; *p* < 0.05 was considered statistically significant.

### 4.3. Animal Study

All animal experimental procedures were approved by the Experimental Animal Academic Ethics Committee of South China University of Technology (AEC2021059). 5-week-old male nude mice were purchased from the GemPharmatech Co., Ltd. (Nanjing, Jiangsu, China), kept in the Laboratory Animal Center of South China University of Technology (Guangzhou, Guangdong, China), and maintained in specific pathogen-free conditions with a stationary temperature of 23–25 °C and 12 h light/dark cycles.

To establish a tumor metastasis model in animals, 5-8F cells were transfected with lentivirus-vector expressing system LV-luciferase (Genechem, Shanghai, China) and selected for stabilized expressing clones by series dilution selection. Mice were randomized into several groups. Then, 1.5 × 10^6^ 5-8F-luc cells were washed and resuspended in 100 μL PBS. Subsequently, cells were intravenously injected into nude mice. Tumor cell metastasis was monitored using the IVIS Lumina series Ⅲ imaging system (Xenogen, Alameda, CA, USA). After 7 weeks, the mice were sacrificed, and the lungs were separated, weighted, and photographed and subsequently fixed and embedded in paraffin before processing for hematoxylin and eosin (HE) and immunohistochemistry staining.

For AdipoRon administration, the mice were randomly assigned into two groups (vehicle and AdipoRon), with 7 mice per group after injection of NPC cells. In the AdipoRon group, mice were intragastrically administered 50 mg/kg AdipoRon suspended in corn oil once a day. In the vehicle group, mice were administered solvent alone in corn oil.

### 4.4. Cell Culture and Regents

The S18 cell line was kindly gifted by Professor Chaonan Qian at Sun Yat-sen University Cancer Center (SYSUCC). The 5-8F cells were from the Advanced Research Center of Central South University (Changsha, Hunan, China). The 5-8F cells were cultured in RPMI-1640 medium, and the S18 cells were cultured in Dulbecco’s modified eagle medium containing 4.5 mg/mL glucose, all supplemented with 10% fetal bovine serum (Gibco, Carlsbad, CA, USA), 100 U/mL penicillin, and 100 ug/mL streptomycin (Hyclone, Logan, UT, USA). The cells were maintained in a humidified atmosphere of 5% CO_2_ at 37 °C. The cell lines were authenticated via deoxyribonucleic-acid profiling using short tandem repeat analysis.

Recombination human adiponectin was dissolved in deionized water to prepare a working stock solution of approximately 0.5 mg/mL (BioVendor, Brno, Czech Republic). Recombination human leptin, IL-6, and TNF-α were dissolved in deionized water to prepare a working stock solution of approximately 0.01 mg/mL (PeproTech, Rocky Hill, NJ, USA). AdipoRon was dissolved in DMSO to prepare a working stock solution of approximately 50 mM (AdooQ, Irvine, CA, USA).

### 4.5. Wound-Healing Assay

The cells were seeded in 12-well plates, and cell confluence was 100% after adherent. Monolayer cells were washed with phosphate-buffered saline (PBS) and scraped with a plastic 200 µL pipette tip, and then incubated with fresh medium treated with resistin. The “wounded” were photographed by microscope at 0 h and 24 h. The relative migration rates were calculated by cell-covered area (0 h)/cell-covered area (24 h).

### 4.6. Migration and Invasion Assays

In the study, 24-well Transwell inserts (BD Biosciences, San Jose, CA, USA) coated with or without growth factor-reduced Matrigel (Corning Incorporated, Corning, NY, USA) were used for migration and invasion assays. NPC cells were pretreated with adiponectin for 48 h. NPC cells were suspended in 200 μL serum-free medium treated with or without adiponectin, added to the upper chamber of a Transwell chamber in duplicate, and incubated for 24 h at 37 °C, allowing them to migrate into the lower chamber containing the medium with 10% FBS. After 24 h of incubation, membrane-trapped cells were fixed, stained with crystal violet, and counted using a light microscope.

### 4.7. Transient Transfection with Small Interfering RNA (siRNA)

The small interfering RNA (siRNA) oligos against AdipoR1, AdipoR2, and scrambled control siRNA were commercially synthesized by RiboBio (Guangzhou, Guangdong, China) and transfected with riboFECT CP transfection reagent (RiboBio, Guangzhou, Guangdong, China) according to the manufacturer’s protocol. The siRNA sequences were as follows: AdipoR1 siRNA#1 sense 5′-CACCGTCTATTGTCATTCA-3′, AdipoR1 siRNA#2 sense 5′-TCCCTGACTGGCTAAAGGA-3′, AdipoR1 siRNA#3 sense 5′-AGAAGGGCAAACGGGTAAT-3′, AdipoR2 siRNA#1 sense 5′-ACTGGATGGTACACGAAGA-3′, AdipoR2 siRNA#2 sense 5′-TCATTCCTACCTTGCACTA-3′, and AdipoR2 siRNA#3 sense 5′-TTATATGTTTCGCCCAAAT-3′.

### 4.8. RNA Extraction and qRT-PCR

Total RNA was isolated from cell by using Trizol reagent (Sigma-Aldrich, St. Louis, MO, USA). cDNA was reversely transcribed using the HiScript II Q RT kit (Vazyme Biotech, Nanjing, China). Quantitative real-time PCR (qRT-PCR) analysis was performed in a qTOWER3 G real-time PCR system (Analytik Jena) by using ChamQ Universal SYBR qPCR Master Mix (Vazyme) according to the manufacturer’s instructions. The relative expression levels of mRNA were normalized to the expression of β-actin by using the 2-ΔΔCT method. Primers were synthesized by Sangon Biotech (Shanghai, China). Sequences of all primers were as follows: ACTB F 5′-CCTGTACGCCAACACAGTGC-3′, R 5′-ATACTCCTGCTTGCTGATCC-3′; AdipoR1 F 5′-ACGTTGGAGGGTCATCCCATA-3′, R 5′-AAACAGCACGAAACCAAGCAG-3′; AdipoR2 F 5′-CCCTCTCTTACAAGCCCATCA-3′, R 5′-GAGCCAGTCTGGTAGTACATCA-3′.

### 4.9. Immunoblotting Analysis

Cells were scraped and lysed with Radio-Immunoprecipitation (RIPA) lysis buffer containing a protease inhibitor (Beyotime Biotechnology, Shanghai, China) and quantified with bicinchoninic acid (BCA) protein assay kit (Thermo Fisher Scientific, Waltham, MA, USA). Nuclear and cytoplasmic protein extraction was analyzed using a Nuclear and Cytoplasmic Protein Extraction Kit (Beyotime) according to the manufacturer’s instructions. Then, the equivalent proteins were separated by SDS-PAGE, and transferred on polyvinylidene fluoride (PVDF) membrane (Millipore, Billerica, MA, USA). The membranes were blocked with 5% nonfat dried milk blocking buffer for 1 h at room temperature, followed by overnight incubation at 4 °C with the primary antibody. Membranes were washed with Tris–HCl buffer containing Tween 20, and then the secondary antibody was incubated for 1 h at room temperature. Blots were detected with ECL detection system (Thermo) using ChemiDoc XRS+ system (Bio-Rad). 

The antibodies used for Western blot analysis included anti-β-Actin (Cat#A2228) and anti-α-Tubulin (Cat#T6074) purchased from Sigma; anti-Histone H3 (Cat#ab1791), anti-MMP-2 (Cat#ab92536), and anti-MMP-9 (Cat#ab58803) purchased from Abcam; anti-Snail (Cat#MA5-14801) purchased from Thermo Fisher Scientific; anti-E-cadherin (Cat#3195), anti-N-cadherin (Cat#13116), anti-Slug (Cat#9585), anti-Claudin-1 (Cat#13255), anti-Vimentin (Cat#5741), anti-STAT3 (Cat#9139), anti-p-STAT3 (Cat#9145), anti-p65 (Cat#8242), anti-p-p65 (Cat#3033), anti-IκBα (Cat#4814), and anti-p-IκBα (Cat#9246) purchased from Cell Signaling Technology; goat antimouse-HRP (Cat#115-035-003) and goat antirabbit-HRP (Cat#111-035-003) purchased from Jackson ImmunoResearch.

### 4.10. Immunofluorescence Staining

NPC cells were plated onto a glass-bottom cell culture dish (Wuxi NEST Biotechnology Co., Ltd., Jiangsu, China). Then, cells were pretreated with adiponectin for two hours before incubation with leptin, IL-6, and TNF-α. Two hours later, cells were permeabilized in PBS containing 0.2% Triton X-100, and then blocked with PBS containing 10% goat serum and 1% BSA (Beyotime) and incubated with primary antibody rabbit anti-p65 (CST; 1:400) and mouse anti-STAT3 (CST; 1:800) in blocking buffer overnight at 4 °C, followed by goat anti-Rabbit Alexa 555 (CST; Cat#4413) fluorescent secondary antibody incubation for 2 h at room temperature. After washing with PBS, cells were mounted with antifade mounting medium with DAPI (Beyotime). The images were obtained using a fluorescent microscope.

### 4.11. Dual-Luciferase Reporter Assay

The pNFκB-luc, pSTAT3-TA-luc, and pRL-TK plasmids were purchased from Beyotime. NPC cells were seeded in BeyoGold™ 96-Well White Opaque Plates (Beyotime) transfected with Lipofectamine™ 3000 Reagent (Invitrogen, Carlsbad, CA, USA). Reporter enzyme activity was determined with a dual-luciferase reporter assay system (Beyotime) according to the manufacturer’s instructions. The luminescence signal was determined with a Varioskan LUX multimode microplate reader (Thermo). Relative luminescence units = Firefly luciferase activity/Renilla luciferase activity.

### 4.12. Immunohistochemistry Staining

IHC was carried out as described previously [33]. The sections were deparaffinized AND rehydrated, and antigen retrieval with the microwave method was performed in a 10 mM citrate buffer. The sections were blocked with 3% H_2_O_2_ for 15 min and incubated with 5% normal goat serum in PBST for 1 h at 37 °C. Then, sections were incubated with primary antibodies rabbit anti-p-p65 (CST; 1:800) and rabbit anti-p-STAT3 (CST; 1:400) at 4 °C overnight. Washing was followed by goat antirabbit-HRP (Jackson ImmunoResearch, West Grove, PA, USA) incubation for 1 h. Sections were incubated with developing solution (diaminobenzidine, DAB) and counterstained with hematoxylin (ZSGB-Bio, Beijing, China).

### 4.13. Statistical Analysis

Data are presented as mean ± SD and were analyzed by Student’s t-test or by analysis of variance (ANOVA) with Sidak’s multiple comparisons test using GraphPad Prism 7.0 (GraphPad Software, La Jolla, CA, USA). A value of *p* < 0.05 was considered statistically significant.

## 5. Conclusions

In summary, the findings here reported for the first time that blood adiponectin level was inversely associated with NPC tumor staging and MFS. Such clinical revelations are further corroborated by our findings that adiponectin can directly inhibit NPC cell migration and invasion through blocking STAT3/NF-κB signaling in NPC cells. Furthermore, pharmacological activation of adiponectin actions can suppress the migration and invasion of NPC cells both in vitro and in vivo. Thus, we tentatively propose the concept that activating adiponectin’s action or elevating blood adiponectin levels may become suitable therapeutic modalities for better treatment of NPC patients. 

## Figures and Tables

**Table 1:** Association between clinical characteristics and serum adiponectin levels in NPC patients.

Variable		*n*	Adiponectin (μg/mL)		Z/*χ*^2^	*p* ^a^
			Median	IQR		
Gender	Female	15	2.17	7.84		
	Male	91	2.12	6.68	−0.567	0.571
Age (years)	≤45	56	2.10	6.14		
	>45	50	2.38	6.68	−0.516	0.606
Grade	I/II	37	3.87	10.81		
	III/IV	69	2.06	5.86	−2.343	0.019
VCA-IgA	≤1:80	26	3.64	8.19		
	>1:80	80	2.12	6.41	−0.958	0.338
EBNA1/IgA	≤20	66	2.32	6.80		
	>20	40	2.13	6.28	−0.404	0.686
Family history	Yes	24	1.67	7.00		
	No	82	2.08	6.09	−2.110	0.035
Recurrence	Yes	5	1.05	4.30		
	No	101	2.28	6.67	−2.161	0.031
Metastasis	Yes	38	1.57	3.47		
	No	68	3.05	8.77	−3.255	0.001
BMI (kg/m^2^)	<25	78	2.59	6.52		
	≥25	28	1.41	3.31	−2.297	0.022

Abbreviations: NPC, nasopharyngeal carcinoma; VCA-IgA, viral capsid antigen-immunoglobulin A; EBNA1/IgA, Epstein–Barr nuclear antigen 1-immunoglobulin A; BMI, body mass index; IQR, interquartile range. Data are presented as median (interquartile range) for adiponectin. ^a^ *p*-values were calculated by Mann–Whitney U test.

**Table 2:** Univariable and multivariable Cox regression analyses of potential factors for the metastasis-free survival in NPC patients ^a^.

Variable	Univariable		Multivariable ^b^	
	*HR (95%CI)*	*p*	*HR (95%CI)*	*p*
Gender				
Female	1.000 (ref)		1.000 (ref)	
Male	1.139 (0.444–2.922)	0.787	0.794 (0.284–2.224)	0.661
Age (years)				
≤45	1.000 (ref)		1.000 (ref)	
>45	0.886 (0.467–1.681)	0.710	0.788 (0.378–1.643)	0.525
Grade				
I/II	1.000 (ref)		1.000 (ref)	
III/IV	22.789 (3.122–166.353)	0.002	23.445 (3.105–177.040)	0.002
VCA-IgA				
≤1:80	1.000 (ref)		1.000 (ref)	
>1:80	1.114 (0.511–2.431)	0.786	0.600 (0.218–1.652)	0.323
EBNA1/IgA				
≤20	1.000 (ref)		1.000 (ref)	
>20	1.518 (0.802–2.871)	0.200	0.934 (0.426–2.050)	0.865
Family history				
No	1.000 (ref)		1.000 (ref)	
Yes	0.912 (0.418–1.991)	0.817	1.074 (0.435–2.653)	0.876
Recurrence				
No	1.000 (ref)		1.000 (ref)	
Yes	3.165 (1.117–8.968)	0.030	1.500 (0.481–4.680)	0.485
BMI (kg/m^2^)				
<25	1.000 (ref)		1.000 (ref)	
≥25	0.727 (0.341–1.547)	0.408	0.507 (0.207–1.241)	0.137
Adiponectin ^c^	0.883 (0.798–0.976)	0.015	0.890 (0.796–0.996)	0.043
Low	1.000 (reference)		1.000 (reference)	
High	0.282 (0.100–0.796)	0.005	0.245 (0.080–0.745)	0.013

Abbreviations: NPC, nasopharyngeal carcinoma; VCA-IgA, viral capsid antigen-immunoglobulin A; EBNA1/IgA, Epstein–Barr nuclear antigen 1–immunoglobulin A; BMI, body mass index; HR, hazard ratio; CI, confidence interval; MFS, metastasis-free survival. ^a^ Univariate and multivariate analyses were performed by Cox proportional hazards’ regression models. ^b^ Multivariable model included age, gender, grade, VCA-IgA, EBNA1/IgA, family history, recurrence, and BMI. ^c^ All subjects were divided into two groups based on serum adiponectin levels with the median value as the cutoff (8.00 μg/mL).

## Data Availability

The data that support the findings of this study are available on request from the corresponding author.

## Supplementary Materials

The following supporting information can be downloaded at: https://www.mdpi.com/article/10.3390/ijms232112729/s1.
