# Supplementary material for: Adiponectin Suppresses Metastasis of Nasopharyngeal Carcinoma through Blocking the Activation of NF-κB and STAT3 Signaling
Source: Int J Mol Sci. 2022 Oct 22;23(21):12729. doi: 10.3390/ijms232112729 (PMC9658954; doi:10.3390/ijms232112729)
Supplement: Supplementary file 1 [file ijms-23-12729-s001.zip › ijms-1933636-supplementary.pdf]

Supplementary Figures

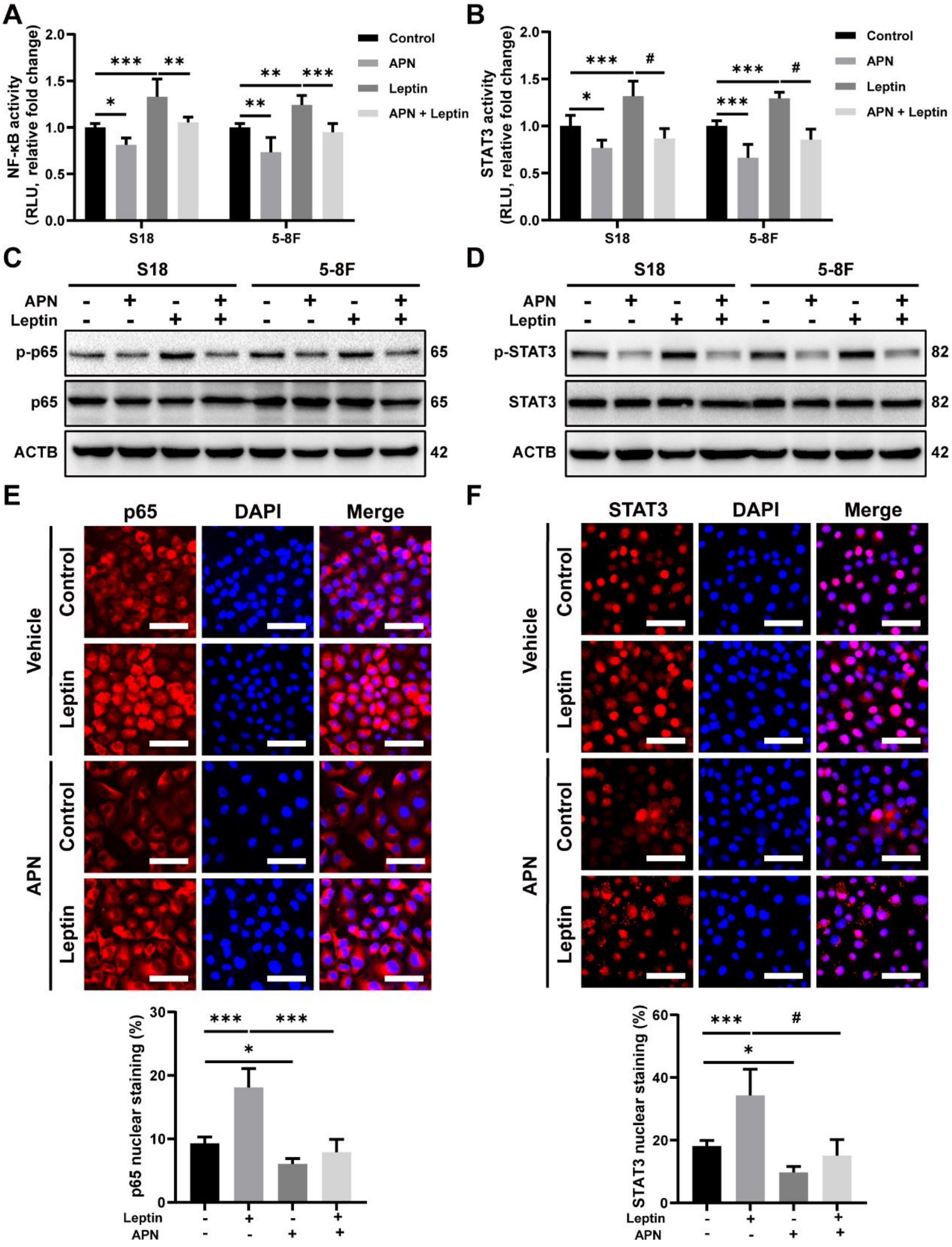

Figure S1. Adiponectin blocks leptin induced the activation of NF-κB and STAT3. (**A**) Luciferase reporter assay of NF-κB in S18 and 5-8F cells pretreated with 40 μg/mL adiponectin for 2 h and followed by 50 ng/mL leptin treatment for 3 h. (**B**) Luciferase reporter assay of

STAT3 in S18 and 5-8F cells pretreated with 40 μg/mL adiponectin for 2 h and followed by 50 ng/mL leptin treatment for 3 h. (**C**) Western blot analysis of p-p65 protein level of S18 and 5-8F cells pretreated with 40 μg/mL adiponectin for 2 h and followed by 50 ng/ml leptin treatment for 30 min. (**D**) Western blot analysis of p-STAT3 protein level of S18 and 5-8F cells pretreated with 40 μg/mL adiponectin for 2 h and followed by 50 ng/mL leptin treatment for 30 min. (**E**) The distribution of NF-κB p65 subunit was visualized by immunofluorescent staining in 5-8F cells pretreated with 40 μg/mL adiponectin for 2 h and followed by 50 ng/mL leptin treatment for 3 h. Scale bar: 20 μm. (**F**) The distribution of STAT3 was visualized by immunofluorescent staining in 5-8F cells pretreated with 40 μg/mL adiponectin and followed by 50 ng/mL leptin treatment for 3 h. Scale bar: 20 μm. Results are presented as mean ± SD of three independent experiments performed in triplicate. $^{*}P<0.05$, $^{**}P<0.01$, $^{***}P<0.001$, $^{\#}P<0.0001$.

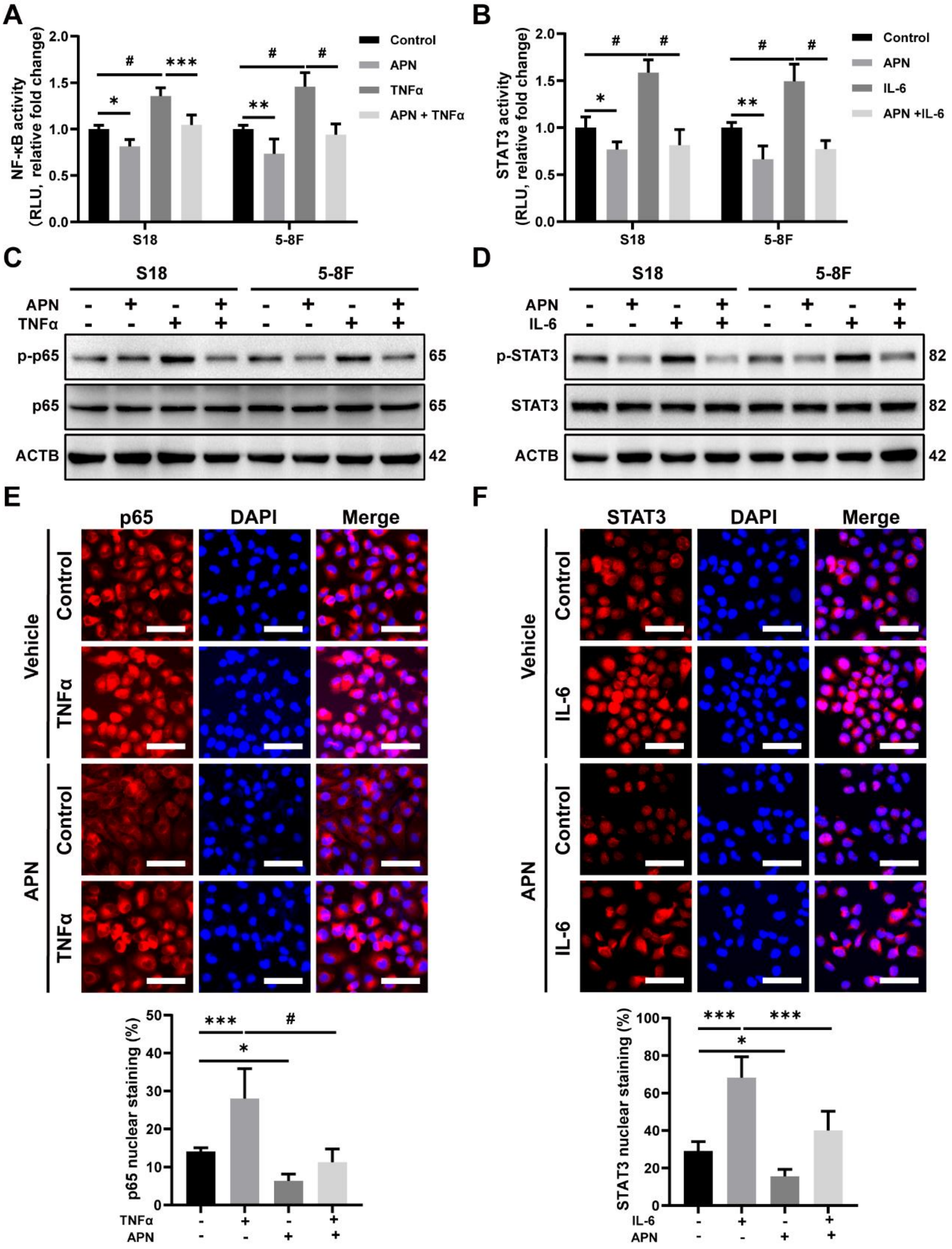

Figure S2. Adiponectin blocks the activation of IL-6 induced STAT3 and TNFα induced NF-κB. (**A**) Luciferase reporter assay of NF-κB in S18 and 5-8F cells pretreated with 40 μg/mL adiponectin for 2 h and followed by 50 ng/mL TNF-α treatment for 3 h. (**B**) Luciferase reporter assay of STAT3 in S18 and 5-8F cells pretreated with 40 μg/mL adiponectin for 2 h and

followed by 50 ng/mL IL-6 treatment for 3 h. (**C**) Western blot analysis of p-p65 protein level of S18 and 5-8F cells pretreated with 40 μg/mL adiponectin for 2 h and followed by 50 ng/mL TNF-α treatment for 30 min. (**D**) Western blot analysis of p-STAT3 protein level of S18 and 5-8F cells pretreated with 40 μg/mL adiponectin for 2 h and followed by 50 ng/mL IL-6 treatment for 30 min. (**E)** The distribution of NF-κB p65 subunit was visualized by immunofluorescent staining in 5-8F cells pretreated with 40 μg/mL adiponectin for 2 h and followed by 50 ng/mL TNF-α treatment for 6 h. Scale bar: 20 μm. (**F**) The distribution of STAT3 was visualized by immunofluorescent staining in 5-8F cells pretreated with 40 μg/mL adiponectin and followed by 50 ng/mL IL-6 treatment for 3 h. Scale bar: 20 μm. Results are presented as mean ± SD of three independent experiments performed in triplicate. **P*<0.05, ***P*<0.01, ****P*<0.001, $^{\#}P$<0.0001.
